# Retinal neural dysfunction in diabetes revealed with handheld chromatic pupillometry

**DOI:** 10.1111/ceo.14116

**Published:** 2022-06-13

**Authors:** Tien‐En Tan, Maxwell T. Finkelstein, Gavin Siew Wei Tan, Anna Cheng Sim Tan, Choi Mun Chan, Ranjana Mathur, Edmund Yick Mun Wong, Chui Ming Gemmy Cheung, Tien Yin Wong, Dan Milea, Raymond P. Najjar

**Affiliations:** ^1^ Singapore Eye Research Institute, Singapore Singapore National Eye Centre Singapore Singapore; ^2^ Duke‐National University of Singapore Medical School Singapore Singapore; ^3^ Department of Ophthalmology, Yong Loo Lin School of Medicine National University of Singapore Singapore Singapore

**Keywords:** diabetic retinopathy, diabetes, melanopsin, photoreceptors, pupillometry, retinal degenerations

## Abstract

**Background:**

To evaluate the ability of handheld chromatic pupillometry to reveal and localise retinal neural dysfunction in diabetic patients with and without diabetic retinopathy (DR).

**Methods:**

This cross‐sectional study included 82 diabetics (DM) and 93 controls (60.4 ± 8.4 years, 44.1% males). DM patients included those without (*n* = 25, 64.7 ± 6.3 years, 44.0% males) and with DR (*n* = 57, 60.3 ± 8.5 years, 64.9% males). Changes in horizontal pupil radius in response to blue (469 nm) and red (640 nm) light stimuli were assessed monocularly, in clinics, using a custom‐built handheld pupillometer. Pupillometric parameters (phasic constriction amplitudes [predominantly from the outer retina], maximal constriction amplitudes [from the inner and outer retina] and post‐illumination pupillary responses [PIPRs; predominantly from the inner retina]) were extracted from baseline‐adjusted pupillary light response traces and compared between controls, DM without DR, and DR. Net PIPR was defined as the difference between blue and red PIPRs.

**Results:**

Phasic constriction amplitudes to blue and red lights were decreased in DR compared to controls (*p* < 0.001; *p* < 0.001). Maximal constriction amplitudes to blue and red lights were decreased in DR compared to DM without DR (*p* < 0.001; *p* = 0.02), and in DM without DR compared to controls (*p* < 0.001; *p* = 0.005). Net PIPR was decreased in both DR and DM without DR compared to controls (*p* = 0.02; *p* = 0.03), suggesting a wavelength‐dependent (and hence retinal) pupillometric dysfunction in diabetic patients with or without DR.

**Conclusions:**

Handheld chromatic pupillometry can reveal retinal neural dysfunction in diabetes, even without DR. Patients with DM but no DR displayed primarily inner retinal dysfunction, while patients with DR showed both inner and outer retinal dysfunction.

## 1 INTRODUCTION

Diabetic retinopathy (DR) is an important microvascular complication of diabetes, and a leading cause of visual impairment and blindness worldwide, particularly among the working‐age population.^1^, ^2^ Research into the retinal manifestations of diabetes has traditionally focused on retinal vascular damage, and indeed, our current clinical classifications of DR focus only on ophthalmoscopically‐visible retinal vascular lesions.^3^, ^4^ However, in the past few years, advances in structural imaging and functional testing have provided mounting evidence of retinal neural abnormalities in diabetes, which have been collectively termed diabetic retinal neurodegeneration (DRN).^5^, ^6^, ^7^, ^8^ Though some of the initial structural imaging studies showed conflicting results, there is now a growing consensus that patients with diabetes can demonstrate thinning in various retinal layers, primarily in the inner retina, such as the retinal nerve fibre layer (RNFL) and ganglion cell layer.^5^, ^6^, ^7^, ^9^, ^10^, ^11^ In many of these studies, structural DRN changes were found in diabetic patients even in the absence of visible DR, which has contributed to the hypothesis that DRN may precede traditionally visible vasculopathy. Various functional retinal abnormalities have also been demonstrated in diabetes, including pattern electroretinogram (PERG) and oscillatory potential (OP) abnormalities, reduced implicit times on multifocal electroretinography (mfERG), reduced contrast sensitivity, and perimetric defects.^12^, ^13^, ^14^, ^15^, ^16^, ^17^, ^18^, ^19^

**FIGURE 1:**
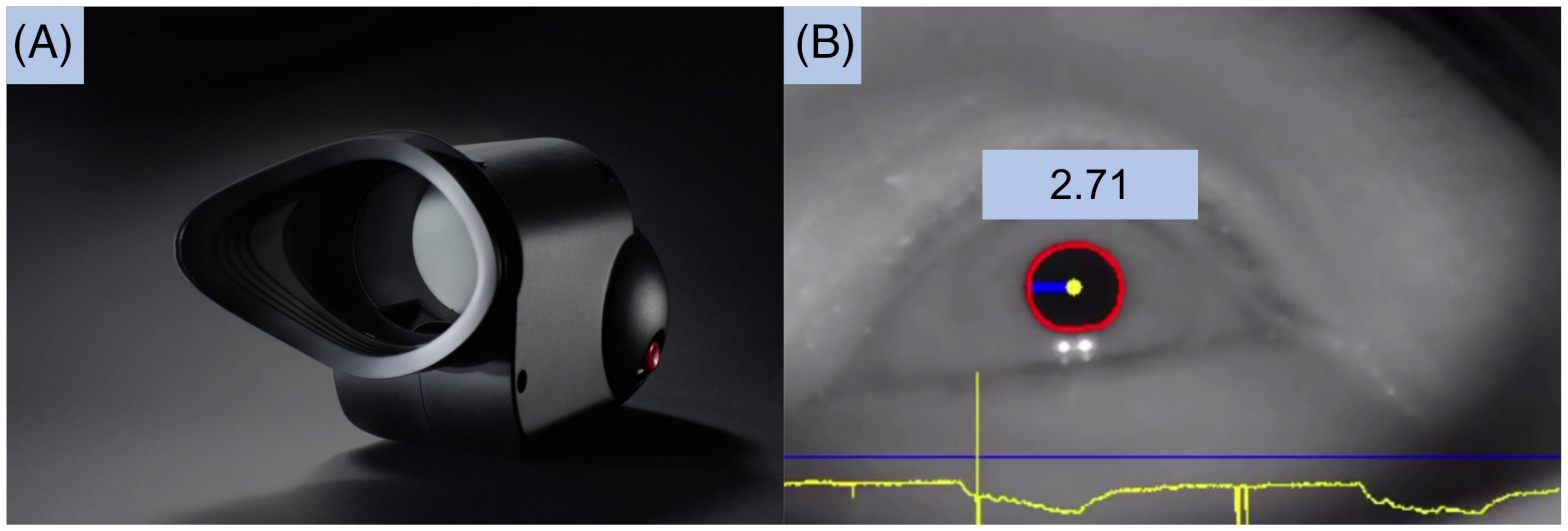
(A) Custom‐built handheld chromatic pupillometer. (B) Infrared camera recording with automated pupil detection (in red), horizontal pupil radius measurement of 2.71 mm, and pupillary trace recording at the bottom of the screen (in yellow)

In addition to rods and cones, the retina also harbours intrinsically photosensitive retinal ganglion cells (ipRGCs).^20^ These cells, expressing the photopigment melanopsin (peak sensitivity ~482 nm), project to multiple image and non‐image forming centres in the brain, including the olivary pretectal nucleus, controlling the pupillary light response (PLR).^21^, ^22^, ^23^ Analysing different aspects of the PLR to chromatic light paradigms allows for evaluation and localization of retinal neural function in health and ocular diseases.^22^, ^24^ For example, phasic constriction amplitudes primarily reflect function of the rods and cones (outer retinal function), while the post‐illumination pupillary response (PIPR), especially in response to blue light stimuli, primarily reflects intrinsic ipRGC function from the inner retina.^20^, ^22^, ^23^ Functional retinal abnormalities have been highlighted using pupillometry in diabetes and DR, with most studies demonstrating dysfunction in the ipRGCs manifesting as abnormalities of the PIPR.^23^, ^25^, ^26^ These studies, however, were performed with large cumbersome Ganzfeld domes in laboratory research settings, which are difficult to translate to routine clinical use. We have recently developed a handheld chromatic pupillometer (HCP; Figure 1) for clinic‐based assessment of the PLR in patients with ocular conditions.^27^, ^28^ The aim of this study was to evaluate the ability of HCP using a standardised 1‐min testing protocol, to reveal and localise retinal neural dysfunction in diabetic patients with and without DR, compared to controls.

## 2 METHODS

This was a cross‐sectional single‐centre study involving 82 participants with diabetes, and 93 healthy controls. Participants with diabetes were recruited from the Diabetic Retinopathy Service at the Singapore National Eye Centre (SNEC) between January 2018 and June 2018. Participants were eligible if they were aged 22 years or older, with either type 1 or type 2 diabetes, and could have a range of DR severity (graded on the International Clinical Diabetic Retinopathy [ICDR] Disease Severity Scale) including no DR, mild non‐proliferative DR (NPDR), moderate NPDR, severe NPDR and proliferative DR.^3^ Key exclusion criteria were: presence of retinal pathology other than DR, previous sectoral or pan‐retinal laser photocoagulation, previous intravitreal anti‐vascular endothelial growth factor (anti‐VEGF) injections, cataract surgery within the past 6 months, previous ocular surgery other than cataract surgery, significant cataract greater than 2+ on the Lens Opacities Classification System III (LOCS III) scale, previous ocular trauma, previous diagnosis of optic neuropathy, uveitis, glaucoma, raised vertical cup‐disc ratio of >0.6, or raised intraocular pressure, and previous diagnosis of autonomic neuropathy, conditions that affect efferent pupillary pathways such as tonic pupils, third cranial nerve palsies or Horner's syndrome, regular use of medications that affect pupillary size or responses, and psychiatric conditions. Healthy controls without diabetes, and with similar exclusion criteria (i.e., no eye diseases or conditions affecting the afferent and efferent pupillary pathways), were recruited from the general ophthalmology clinics of SNEC. Only one eye from each participant was included in this study—in participants where both eyes were eligible, the study eye was assigned at random.

Institutional Review Board (IRB)/Ethics Committee approval was obtained from the SingHealth Centralised IRB, and this study was conducted in accordance with the tenets of the Declaration of Helsinki. All participants provided signed informed consent.

### 2.1 Clinical evaluation and imaging

Demographic data collected from participants included: age, sex, ethnicity, glycated haemoglobin A1c (HbA1c) levels (where available, and only if taken within 3 months of participation in the study), co‐morbid hypertension and ischemic heart disease and smoking history. All participants underwent a thorough ophthalmic evaluation including measurement of best corrected visual acuity (BCVA) (LogMAR chart, Lighthouse Int., NY, USA), intraocular pressure measurement by either non‐contact tonometry or Goldmann applanation tonometry, and slit‐lamp biomicroscopy. Prior to pharmacologic mydriasis, all participants underwent HCP with a standardised 1‐min protocol (see details below). Thereafter, all participants underwent pharmacologic mydriasis, followed by indirect ophthalmoscopy, 45° colour fundus photography of Early Treatment of Diabetic Retinopathy Study (ETDRS) standard fields 1 (optic disc‐centred) and 2 (macula‐centred) (Nonmyd WX^3D^; Kowa Company Ltd, Aichi, Japan) and optical coherence tomography (OCT) of the peripapillary RNFL and macular GCC (Cirrus HD‐OCT 4000; Carl Zeiss Meditec AG, Jena, Germany). Fundus photographs were classified by masked, trained graders from an ocular reading centre, according to the ICDR Disease Severity Scale.^3^ Automated OCT segmentations were used to obtain average peripapillary RNFL and macular ganglion cell complex (GCC) thickness values.

### 2.2 Handheld chromatic pupillometry

All participants underwent the same standardised 1‐min protocol for measurement of direct pupillary responses to chromatic light in the study eye, using a custom‐built handheld chromatic pupillometer in dedicated dark rooms (<1 lux), with the fellow eye occluded. Briefly, the handheld chromatic pupillometer used in this study is a roughly cylindrical, portable, lightweight system (~300 g), that can be easily held by patients during the administration of light stimuli and assessment of the PLR (Figure 1). The device is used monocularly (for either the right or left eye). It has a rounded posterior handgrip for ease of handling by the participant. Its anterior portion holds a moulded silicone rubber eye cup that fits comfortably over a patient's bony orbital rim to allow a stable positioning and light‐isolation of the study eye. The silicone rubber eye cup can be rotated to fit over the right or left eye, and can be detached for sterilisation and cleaning. Other key elements of the device include a light stimulation chamber consisting of a panel of red‐green‐blue [RGB] light emitting diodes [LEDs] coupled with two light diffusers, and an infrared (IR) camera oriented at a ~60° angle below the lower eyelid for direct recording of changes in pupil size of the study eye. The IR camera recorded videos with a spatial resolution of 1280 × 768 pixels at a frame rate of 30 frames per second. Light stimuli of varying wavelength and intensity are delivered by the RGB LEDs through two diffusers to stimulate the central 50° of the visual field (without considering any reflections off the interior of the device). The closest light diffuser to the participant's study eye was located 55–65 mm from the cornea. The light intensity and spectra of the device were calibrated using a calibrated radiometer (ILT5000, International Light Technologies, Peabody, MA, USA) and spectroradiometer (ILT950, International Light Technologies, Peabody, MA, USA) with the light sensor positioned at the subject's eye level. The RGB LEDs and IR camera are controlled using a Raspberry PI Zero‐W single board computer (Raspberry Pi Foundation, UK) and powered by a rechargeable 3.7 V lithium battery. The device is operated remotely via a mobile tablet (iPad Mini, Apple, CA, USA) with a custom‐built iOS application that allows for the control of light stimulation protocols and real‐time monitoring of fixation, blinks, and automated pupil detection. The application also allows for post‐hoc removal of blink artefacts and measurement of horizontal pupil radius as a function of time (Figure 1). Pupillometric traces were stored on the mobile tablet as .csv files.

The standardised HCP protocol used in this study has been previously validated in healthy controls and subjects with glaucoma.^28^ The 1‐min protocol did not include a dark adaptation period, and consisted of five consecutive phases: (1) 10 s of darkness for measurement of baseline pupil size, (2) 9 s of exponentially increasing blue light (11.7–14.4 Log photons/cm^2^/s; peak wavelength [*λ*_max_] = 469 nm, full width at half maximum [FWHM] = 33 nm), (3) 22 s of darkness to pupillary redilation to baseline, (4) 9 s of exponentially increasing red light (11.9–14.3 Log photons/cm^2^/s; *λ*_max_ = 640 nm, FWHM = 17 nm), and (5) 10 s of darkness to assess pupillary redilation. During the stimulation protocol, participants were instructed to fixate on a central dim red zone (<0.1 lux) delivered using an RGB LED. This blurred central red fixation zone was dim (<0.1 lux, <11.0 Log Photons/cm^2^/s), 10°–12° in size, discoidal in shape, and did not elicit any accommodation reflex or PLR.^29^ Poor fixation or excessive blinking were identified in real‐time, and the HCP testing was repeated whenever necessary.

### 2.3 Statistical analysis

**FIGURE 2:**
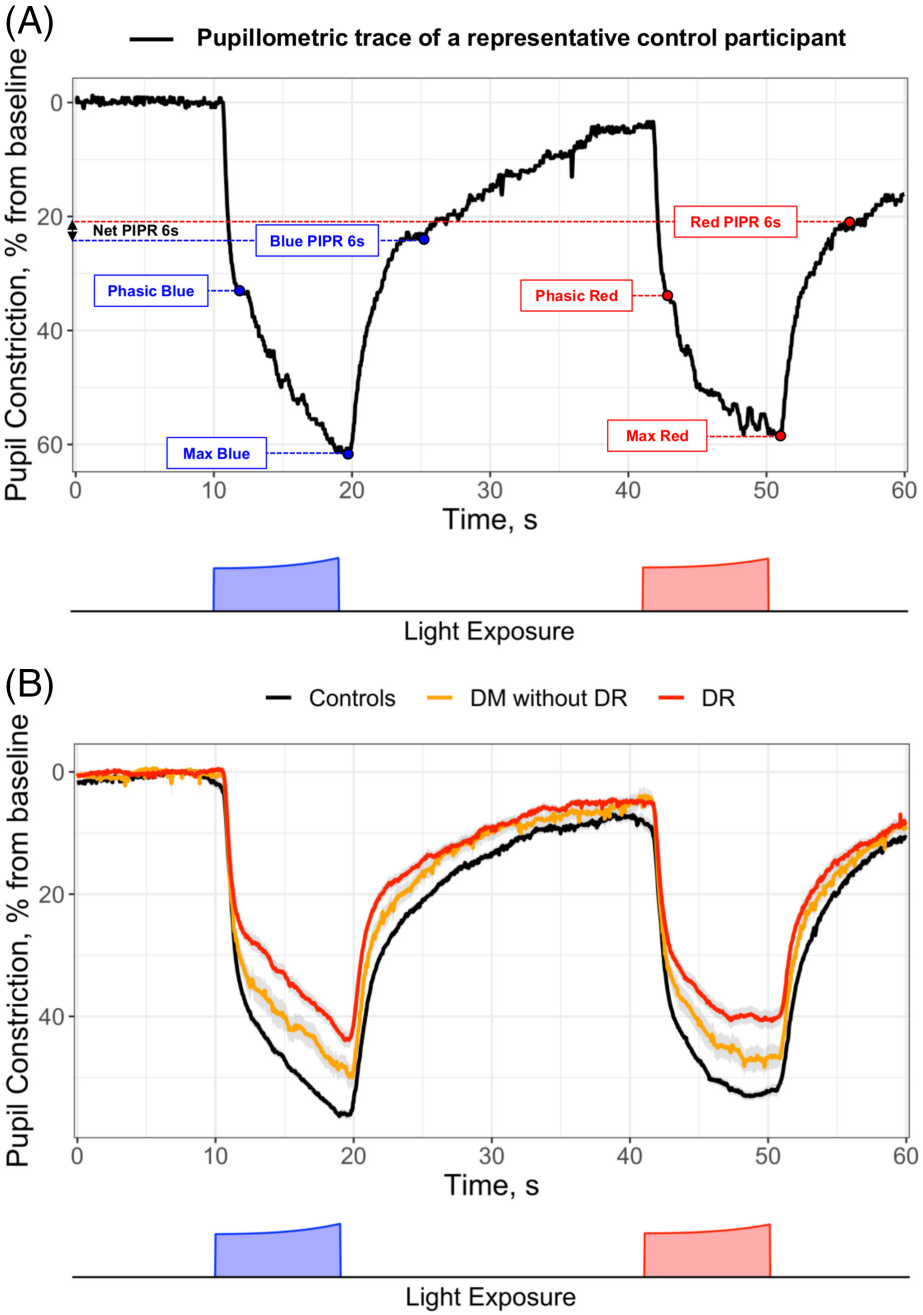
Baseline‐adjusted pupillary responses to blue and red lights over a standardised 1‐min protocol in controls and patients with diabetes (DM), with and without diabetic retinopathy (DR). (A) shows an example pupillary constriction trace for a representative control participant, with the measurement of various pupillometric parameters marked on the figure with dotted lines. (B) shows average pupillary traces as mean ± standard error for controls (black line), DM without DR (yellow line), and DR (red line). The 1‐min protocol (depicted at the bottom of each figure) consists of 10 s of darkness for measurement of baseline pupil size, 9 s of exponentially increasing blue light (11.7–14.4 Log photons/cm^2^/s; peak wavelength [*λ*_max_] = 469 nm, full width at half maximum (FWHM) = 33 nm), 22 s of darkness to pupillary redilation to baseline, 9 s of exponentially increasing red light (11.9 to 14.3 Log photons/cm^2^/s; *λ*_max_ = 640 nm, FWHM = 17 nm), and 10 s of darkness to assess pupillary redilation. Phasic, median pupil constriction 0.5–2.5 s following blue or red light onset; Max, maximal constriction amplitude to blue or red light; PIPR 6 s, post‐illumination pupillary response assessed as the median constriction amplitude 5–7 s after blue or red light offset; net PIPR 6 s, difference between blue PIPR 6 s and red PIPR 6 s. DM, diabetes mellitus; DR, diabetic retinopathy; s, seconds

Pupillometric parameters were extracted from the pupillary response traces of each study eye. Baseline pupil size was defined as the median horizontal radius of the pupil during the 5 s prior to blue light stimulus onset. All subsequent pupillary traces and measurements were expressed as percent constriction from baseline (Figure 2). This accounted for inter‐individual variability in baseline pupil size. Seven pupillometric parameters were extracted (Figure 2): (1) phasic blue = median pupil constriction 0.5–2.5 s following blue light onset; (2) phasic red = median pupil constriction 0.5–2.5 s following red light onset; (3) max blue = maximal constriction amplitude to blue light; (4) max red = maximal constriction amplitude to red light; (5) blue PIPR 6 s = post‐illumination pupillary response assessed as the median constriction amplitude 5–7 s after blue light offset; (6) red PIPR 6 s = post‐illumination pupillary response assessed as the median constriction amplitude 5–7 s after red light offset; and (7) Net PIPR 6 s = difference between blue PIPR 6 s and red PIPR 6 s. These features, along with demographic and clinical characteristics of participants were compared between controls, diabetic patients without DR (‘DM without DR’), and diabetic patients with DR (‘DR’). For categorical variables, differences between groups were assessed using a Pearson's Chi‐squared test followed by post hoc pairwise Chi‐squared tests, or a Fisher's exact test followed by post hoc pairwise Fisher tests. For continuous variables, normal and homoscedastic data were compared using one‐way ANOVA followed by post‐hoc Tukey tests. Normal and heteroscedastic data were compared using Welch's ANOVA and post‐hoc Games–Howell tests, while non‐normal data were compared using Kruskal–Wallis one‐way ANOVA and post‐hoc Dunn's tests. Linear regression was used to assess differences between groups when age was identified as a significant covariate. Correlations between pupillometric parameters and OCT thickness measurements were examined using Pearson's product moment correlation coefficient, with Benjamini‐Hochberg correction for multiple comparisons. For comparison of pupillometric parameters stratified by glycemic control, Student's *t*‐test was used for normal and homoscedastic data, while Welch's *t*‐test was used for heteroscedastic data. Linear regression was used when age was identified as a significant covariate. The threshold for significance for all statistical tests was set at *α* = 0.05, and all post‐hoc tests employed Benjamini‐Hochberg correction for multiple comparisons.

As an exploratory analysis, we evaluated the ability of HCP to (1) classify diabetic patients without or with DR (DM without DR, and DR) from controls; (2) classify patients without DR (controls, and DM without DR) from patients with DR, and (3) classify diabetic patients without DR (DM without DR) from diabetic patients with DR. For each analysis, we used Gradient Boosting Machine methods (GBMs) with 10‐fold cross validation to determine the optimal pupillometric parameters for discrimination, and then built logistic generalised linear models (GLMs) using these parameters for the aforementioned classifications.^28^ Classification performances were assessed using receiver operating characteristic (ROC) analyses. Classification performance outcomes included the area under the receiver operating characteristic curve (AUC), sensitivity and specificity. Optimum classification cutoffs were selected using the highest Youden's J. Confidence intervals were estimated using DeLong's method for AUCs, and a bootstrapping procedure (*n* = 2000) for sensitivity and specificity. Machine learning and statistical procedures were performed using R V.3.6.3: A Language and Environment for Statistical Computing (R Core Team, Vienna, Austria).

## 3 RESULTS

This study included 82 participants with diabetes, of which 25 had no DR (‘DM without DR’ group; mean age 64.7 ± 6.3 years, 44.0% male, 80.0% of Chinese ethnicity) and 57 had DR (‘DR’ group; mean age 60.3 ± 8.5 years, 64.9% male, 66.7% of Chinese ethnicity). All 82 diabetic individuals had type 2 diabetes. Of the 57 participants with DR, 34 (59.6%) had mild NPDR, 21 (36.8%) had moderate NPDR, and 2 (3.5%) had severe NPDR. We also included 93 healthy controls (mean age 60.4 ± 8.4 years, 44.1% male, 92.5% of Chinese ethnicity). Baseline demographic and clinical characteristics of controls, DM without DR and DR groups are shown in Table 1. Age was not significantly different between groups (*p* > 0.05). However, age was a significant covariate for baseline pupil size, phasic red and max blue, and so was accounted for in these statistical calculations by linear regression. In terms of sex, there was a significant difference found overall by omnibus Chi‐squared test (*p* = 0.04), but no pairwise comparisons reached significance after correction for multiple comparisons. Other baseline characteristics, other than ethnicity (*p* < 0.001) and BCVA (*p* < 0.001), were not significantly different between controls, DM without DR, and DR groups. In particular, mean HbA1c (7.2 ± 1.0%, *n* = 10 vs. 7.6 ± 1.2%, *n* = 47; *p* = 0.21) and the proportion of individuals with poor glycemic control (defined as HbA1c ≥ 8.0%; 20.0%, *n* = 10 vs. 36.2%, *n* = 47; *p* = 0.47) were similar between DM without DR and DR groups. There were also no significant differences in mean peripapillary RNFL thickness (*p* = 0.42) or macular GCC thickness (*p* = 0.72) across the 3 groups.

**TABLE 1:** Demographic and clinical characteristics of the study groups

Demographic and clinical characteristics	Controls (*n* = 93)	DM		*p* Value
		No DR (*n* = 25)	DR (*n* = 57)	
Age, years, mean (SD)^a^	60.4 (8.4)	64.7 (6.3)	60.3 (8.5)	0.054
Sex, no. male (%)^b^	41 (44.1)	11 (44.0)	37 (64.9)	0.04**
Ethnicity^c^				<0.001*
Malay, no. (%)	1 (1.1)	1 (4.0)	9 (15.8)	
Indian, no. (%)	1 (1.1)	2 (8.0)	9 (15.8)	
Chinese, no. (%)	86 (92.5)	20 (80.0)	38 (66.7)	
Others, no. (%)	5 (5.4)	2 (8.0)	1 (1.8)	
HbA1c, %, mean (SD)^d^	‐	7.2 (1.0) [*n* = 10]	7.6 (1.2) [*n* = 47]	0.21
Poor glycemic control, HbA1c ≥ 8.0%, no. (%)^c^	‐	2 (20.0) [*n* = 10]	17 (36.2) [*n* = 47]	0.47
HTN, no. (%)^b^	26 (28.9)	11 (44.0)	14 (24.6)	0.20
IHD, no. (%)^c^	6 (6.7)	1 (4.0)	3 (5.3)	1
Smoking history, no. (%)^c^	7 (7.8)	2 (8.0)	1 (1.8)	0.25
BCVA, logMAR, mean (SD)^a^	0.1 (0.2)	0.2 (0.2)	0.2 (0.2)	<0.001*
OCT				
Peripapillary RNFL thickness, μm, mean (SD)^e^	95.4 (8.8)	93.1 (7.2)	94.1 (12.4)	0.42
Macular GCC thickness, μm, mean (SD)^a^	79.0 (8.4)	80.6 (5.1)	79.1 (10.7)	0.72

Abbreviations: DM, diabetes mellitus; DR, diabetic retinopathy; HbA1c, glycated haemoglobin A1c; HTN, hypertension; IHD, ischemic heart disease; BCVA, best‐corrected visual acuity; logMAR, logarithm of minimum angle of resolution; OCT, optical coherence tomography; RNFL, retinal nerve fibre layer; GCC, ganglion cell complex.

^a^ Assessed using One‐way ANOVA followed by post hoc Tukey tests.

^b^ Assessed using Pearson's Chi‐squared test followed by post hoc pairwise Chi‐squared tests with Benjamini‐Hochberg correction.

^c^ Assessed using Fisher's exact test followed by post hoc pairwise Fisher tests with Benjamini‐Hochberg correction.

^d^ Assessed using Mann–Whitney *U* test.

^e^ Assessed using Welch's ANOVA followed by post hoc Games–Howell tests.

* Significant difference between Controls versus DR.

** Significant difference found by omnibus Chi‐squared test, but no pairwise comparisons reached significance after *p*‐value correction for multiple comparisons.

### 3.1 Pupillometric differences between controls, DM without DR, and DM with DR

**FIGURE 3:**
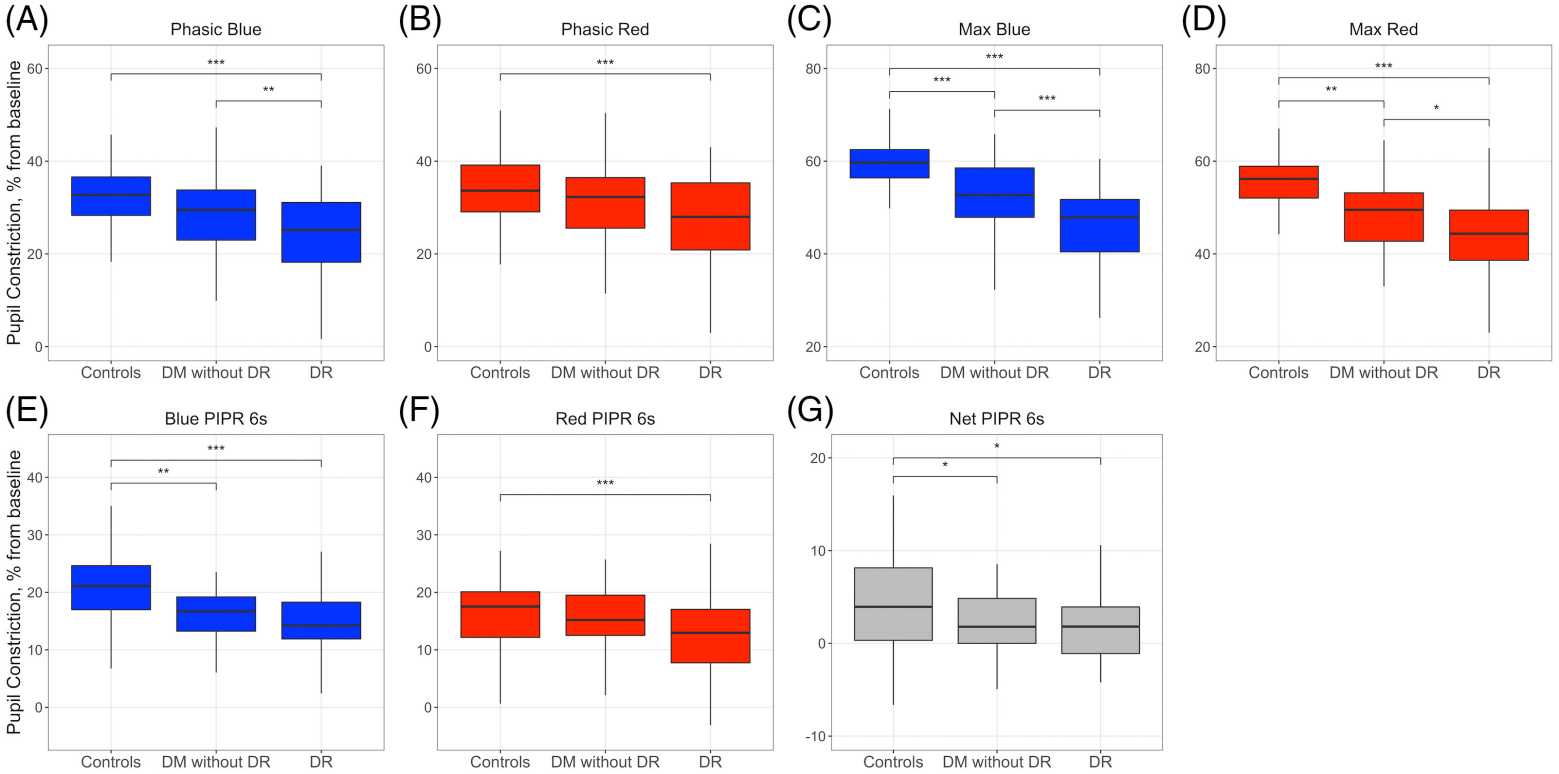
Pupillometric parameters in controls and patients with diabetes, with and without diabetic retinopathy. Differences between groups in phasic (A, B) and maximal pupillary constriction amplitudes (C, D), PIPR 6s (E, F), and Net PIPR 6s (G), in response to the blue (469 nm) and red (640 nm) light stimuli. DM, diabetes mellitus; DR, diabetic retinopathy. Difference between groups is indicated as * for *p* < 0.05, ** for *p* < 0.01, and *** for *p* < 0.001.

Average baseline‐adjusted PLR traces were distinct between controls, DM without DR and DR (Figure 2). Baseline pupil size (i.e., horizontal pupil radius) was smaller in DR (2.6 ± 0.6 mm) compared to controls (3.2 ± 0.6 mm, *p* < 0.001) and compared to DM without DR (3.0 ± 0.7 mm, *p* = 0.008). Twenty‐two seconds after blue light offset, and before onset of the red light stimulus, 80% of subjects had returned to within 10% of the baseline pupil size, which is in line with other studies using the same chromatic pupillometry protocol.^28^, ^29^ Multiple pupillometric parameters were reduced in DM without DR compared to controls, including max blue (*p* < 0.001), max red (*p* = 0.005), blue PIPR 6 s (*p* = 0.004) and net PIPR 6 s (*p* = 0.03) (Table 2, Figure 3). These differences remained significant even when considering only a subgroup of patients with controlled diabetes (Table S1). Various pupillometric parameters were reduced in DR compared to DM without DR, including phasic blue (*p* = 0.01), max blue (*p* < 0.001) and max red (*p* = 0.02) (Table 2, Figure 3). All seven pupillometric parameters were significantly reduced in DR compared to controls. Net PIPR 6 s was decreased in both DR and DM without DR compared to controls (*p* = 0.02; *p* = 0.03), but similar between DM without DR and DR (*p* = 0.92), highlighting a clear wavelength‐dependent retinal alteration in patients with diabetes, with or without DR (Table 2, Figure 3). Recognising that, by 22 s of darkness, not all pupils would have returned to the original baseline prior to blue light onset, we conducted additional analyses where pupillary responses to the red light stimulus were adjusted to the baseline pupil size before red light stimulus onset. These results are presented in the Table S2. The difference in red PIPR 6 s between DR and controls lost statistical significance, but otherwise there were no meaningful differences in results. No significant correlations were found between peripapillary RNFL or macular GCC thicknesses and pupillometric parameters.

**TABLE 2:** Pupillometric parameters in controls and patients with diabetes, with and without diabetic retinopathy

Pupillometric parameters	Controls (*n* = 93)	DM		*p* Value
		No DR (*n* = 25)	DR (*n* = 57)	
Baseline pupil size, mm, mean (SD)^a^	3.2 (0.6)	3.0 (0.7)	2.6 (0.6)	<0.001** ***
Phasic blue, %, mean (SD)^b^	32.6 (6.9)	29.6 (9.4)	24.0 (8.5)	<0.001** ***
Phasic red, %, mean (SD)^a^	33.8 (7.7)	30.6 (9.0)	27.6 (8.8)	<0.001**
Max blue, %, mean (SD)^a^	58.9 (5.2)	51.3 (8.6)	45.2 (9.9)	<0.001* ** ***
Max red, %, mean (SD)^c^	55.6 (6.1)	48.8 (9.0)	42.4 (10.2)	<0.001* ** ***
Blue PIPR 6 s, %, mean (SD)^b^	21.1 (6.4)	16.4 (6.8)	14.2 (6.5)	<0.001* **
Red PIPR 6 s, %, mean (SD)^b^	16.6 (6.1)	14.4 (6.3)	12.6 (7.0)	<0.001**
Net PIPR 6 s, %, mean (SD)^c^	4.5 (5.8)	2.0 (3.2)	1.7 (5.3)	0.004* **

Abbreviations: DM, diabetes mellitus; DR, diabetic retinopathy.

^a^ Assessed using linear regression with age as a covariate.

^b^ Assessed using One‐way ANOVA followed by post hoc Tukey tests.

^c^ Assessed using Welch's ANOVA followed by post hoc Games–Howell tests.

* Significant difference between Controls versus No DR.

** Significant difference between Controls versus DR.

*** Significant difference between No DR versus DR.

### 3.2 Pupillometric differences between controlled and uncontrolled diabetes

Patients with uncontrolled diabetes (HbA1c ≥ 8.0%, *n* = 19) had significant reductions in baseline pupil size (*p* = 0.01), max blue (*p* = 0.02) and max red (*p* = 0.01) compared to patients with controlled diabetes (HbA1c < 8.0%, *n* = 38). The distribution of patients with and without DR was not significantly different between the two groups (*p* = 0.47, Table 1). Pupillometric parameters in patients with controlled and uncontrolled diabetes are shown in Table 3. We also conducted secondary analyses where pupillary responses to the red light stimulus were adjusted to the baseline pupil size before red light stimulus onset, and the results were similar. These results are presented in Table S3.

**TABLE 3:** Pupillometric parameters in patients with diabetes, stratified based on glycemic control

Pupillometric parameters	HbA1c < 8.0% (*n* = 38)	HbA1c ≥ 8.0% (*n* = 19)	*p* Value
Baseline pupil size, mm, mean (SD)^a^	2.9 (0.6)	2.4 (0.6)	0.01
Phasic blue, %, mean (SD)^a^	23.9 (7.5)	25.0 (9.9)	0.65
Phasic red, %, mean (SD)^a^	28.0 (8.4)	26.4 (9.8)	0.54
Max blue, %, mean (SD)^a^	48.2 (8.0)	41.8 (11.5)	0.02
Max red, %, mean (SD)^a^	45.0 (8.8)	38.1 (10.7)	0.01
Blue PIPR 6 s, %, mean (SD)^a^	14.6 (6.0)	12.5 (8.2)	0.28
Red PIPR 6 s, %, mean (SD)^b^	13.2 (5.8)	10.6 (9.3)	0.28
Net PIPR 6 s, %, mean (SD)^c^	1.4 (2.9)	1.9 (7.8)	0.72

Abbreviations: HbA1c, glycated haemoglobin A1c.

^a^ Assessed using Student's *t*‐test.

^b^ Assessed using Welch's *t*‐test.

^c^ Assessed using linear regression with age as a covariate.

### 3.3 Classification performance of HCP

**FIGURE 4:**
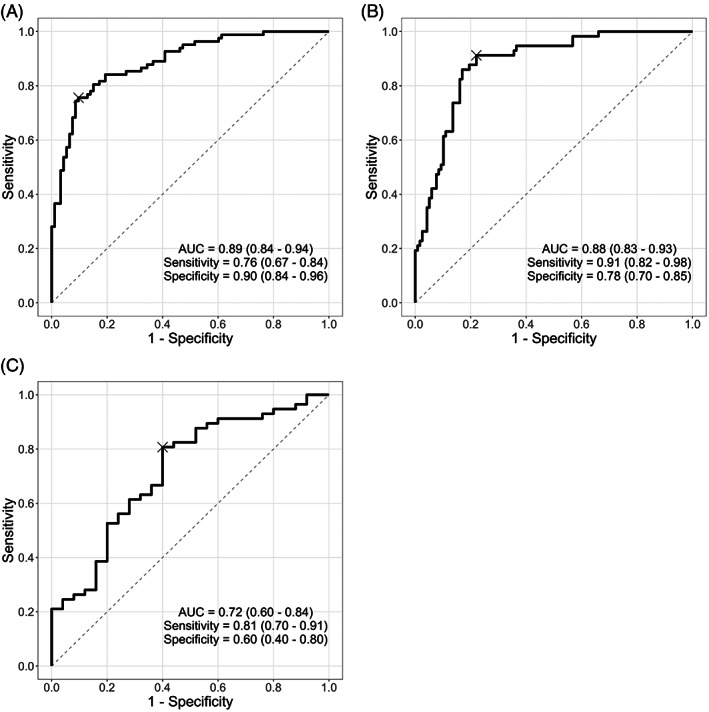
Receiver operating characteristic (ROC) curves used to assess the classification performance of handheld chromatic pupillometry. Generalised linear models were built using only pupillometric parameters to distinguish: (A) controls (*n* = 93) versus diabetic patients without or with diabetic retinopathy (DM without DR, and DR; *n* = 82), (B) patients without DR (controls, and DM without DR; *n* = 118) versus patients with DR (*n* = 57), and (C) diabetic patients without DR (DM without DR; *n* = 25) versus diabetic patients with DR (*n* = 57). Optimum classification cutoffs were selected using the highest Youden's J, and are indicated on the ROC curves with a cross. The area under the ROC curve (AUC), as well as the sensitivity and specificity at optimum cutoff are indicated for each ROC analysis, along with associated 95% confidence intervals.

For the classification of diabetic patients without or with DR (DM without DR, and DR; *n* = 82) from controls (*n* = 93), HCP yielded an AUC of 0.89 (95% confidence interval [CI]: 0.84–0.94), and a sensitivity and specificity of 0.76 (0.67–0.84) and 0.90 (0.84–0.96), respectively. For the classification of patients without DR (controls, and DM without DR; *n* = 118) from patients with DR (*n* = 57), the AUC was 0.88 (0.83–0.93), while the sensitivity and specificity of HCP were 0.91 (0.82–0.98) and 0.78 (0.70–0.85), respectively. For the classification of diabetic patients without DR (DM without DR; *n* = 25) from diabetic patients with DR (*n* = 57), the AUC was 0.72 (0.60–0.84), while the sensitivity and specificity of HCP were 0.81 (0.70–0.91) and 0.60 (0.40–0.80), respectively. Pupillometric parameters used in the classification models above are detailed in Figure S1. To confirm that our findings were not affected by overfitting, we performed additional AUC analyses using 5‐fold cross‐validation. These analyses produced similar results with AUC values of 0.89 (0.85–0.94), 0.88 (0.83–0.93), and 0.76 (0.65–0.87), for the three classification tasks, respectively. ROC curves for the three classification tasks are shown in Figure 4.

## 4 DISCUSSION

In this study, we demonstrate evidence of retinal neural dysfunction in diabetic patients, even without clinically visible DR, using a 1‐min‐long chromatic pupillometry protocol delivered using a portable, lightweight, handheld device. Diabetes can affect pupillary responses to light through either the afferent (retinal) or efferent (parasympathetic) pathways. However, since the pupillometric abnormalities reported in our study are wavelength‐dependent (net PIPR 6 s, which represents the difference between blue and red PIPR 6 s, was decreased in both DR and DM without DR compared to controls), this points to significant functional retinal abnormalities in diabetes. While pupillometric responses can already be impaired in diabetics without DR, patients with DR in our study showed greater dysfunction, with phasic and maximal constrictions to blue light, and maximal constriction to red light all significantly reduced in patients with DR compared to patients without DR. In a subset analysis, we also found that patients with poor glycemic control (HbA1c ≥ 8.0%) also had greater pupillometric dysfunction, independent of DR status. Furthermore, in an exploratory analysis, we demonstrate that HCP has potential for classification of individual patients with diabetes or DR, which may have utility in specific ophthalmic or non‐ophthalmic clinical settings.

Our study, the largest to date examining chromatic pupillometry in diabetes and/or DR, provides valuable contribution to the literature surrounding the pathophysiology of diabetes, DR and associated pupillometric/ocular dysfunction. Only a few published studies have examined chromatic pupillometry in patients with diabetes and DR.^23^, ^25^, ^26^, ^30^ Most of these studies had relatively modest sample sizes, and were performed using large Ganzfeld domes or desk‐bound pupillometers in laboratory‐based settings. Here, we employ a convenient, handheld pupillometry device with a short 1‐min testing protocol, which may be better suited to clinical translation. In addition, compared to previous studies, our study included a significant number of diabetic patients without DR, which allowed us to clearly demonstrate retinal neural dysfunction in the absence of clinically visible vascular changes, and examine the differences between diabetics with and without DR.^23^, ^25^, ^26^ A few large pupillometry studies, using white flashes of light, have demonstrated pupillometric abnormalities in diabetes.^31^, ^32^, ^33^, ^34^, ^35^ However, given the nature of their lighting stimuli, these studies were unable to attribute the detected pupillometric abnormalities to retinal or afferent dysfunction, which led to the conclusion that abnormalities observed were primarily related to autonomic nervous system dysfunction along the efferent pupillary pathway. Other strengths of our study include a “clean” dataset that was ocular treatment‐naïve, without any previous sectoral or pan‐retinal laser photocoagulation or previous intravitreal anti‐VEGF injections, and with standardised grading of DR by masked, trained graders from an ocular reading centre.

Analysis of the specific pupillometric features that are altered allows us to draw some inferences on the potential sites of retinal dysfunction. In our study, diabetic patients without DR showed significant reductions in the maximal constrictions to blue and red lights, but also in the PIPR in response to blue light and net PIPR compared to controls. The PIPR, especially in response to blue light, is largely mediated by the intrinsic aptitudes of ipRGCs, expressing the photopigment melanopsin, with a peak spectral sensitivity around 480 nm.^22^, ^23^, ^36^ Furthermore, the reduction in the amplitude of the net PIPR 6 s further confirms that the dysfunction observed in DM without DR is predominantly at the level of the ipRGCs in the inner retina. These findings are consistent with animal studies, and other published pupillometry studies in diabetic patients.^23^, ^25^, ^26^, ^37^, ^38^ On the other hand, diabetic patients without DR also did not show any significant reductions in phasic constrictions compared to controls. These phasic and transient pupillary responses to light are predominantly driven by outer retinal photoreceptors (rods and cones).^20^, ^23^ Compared to diabetic patients without DR, diabetic patients with DR showed a significant reduction in the phasic pupillary constriction to blue light, as well as a reduction in the maximal constriction amplitudes to blue and red lights. Maximal constriction amplitudes (max blue/red) likely represent mixed contributions from both inner and outer retinal elements. These findings on chromatic pupillometry suggest that diabetics without DR exhibit primarily inner retinal dysfunction, while diabetics with DR seem to display both inner and outer retinal dysfunction, which are consistent with conclusions from other functional studies such as full‐field electroretinography (ffERG), PERG, and mfERG.^39^, ^40^ However, a prospective, longitudinal study would be needed to firmly establish a temporal relationship between inner and outer retinal dysfunction in diabetes. It would also be interesting to investigate in future studies if the inner retinal dysfunction in diabetes is related to impairment in inner retinal perfusion that can be quantified on fluorescein angiography or OCT angiography. It is also worth noting that both inner retinal dysfunction (particularly of ipRGCs) and outer retinal dysfunction (of rod photoreceptors) have been linked to circadian rhythm dysfunction and sleep abnormalities in patients with diabetes.^26^, ^41^ Experimental work has been conducted on supplemental light exposure and light therapy in diabetes, and it would be interesting to examine the relationship between such treatment, sleep architecture, and chromatic pupillometry responses in future studies.^42^, ^43^

Despite the significant inner retinal dysfunction demonstrated using HCP, in our study these functional abnormalities were not mirrored by structural OCT abnormalities of the RNFL or GCC. In fact, here we report no thinning of mean peripapillary RNFL or macular GCC in the OCT measurements between DR, DM without DR or control groups (Table 1). Furthermore, there were no significant associations between pupillometric parameters and OCT metrics. In contrast, pupillometric studies in glaucoma have demonstrated strong correlations between pupillometric abnormalities and OCT parameters.^29^, ^44^ Studies examining RNFL and GCC thickness in diabetes and DR have shown some conflicting results, with some studies demonstrating significant thinning in diabetes and DR compared to controls, while others do not.^9^ However, based on longitudinal studies, meta‐analyses and systematic reviews of the literature, there is a growing consensus that patients with diabetes and DR can demonstrate retinal thinning on OCT.^6^, ^9^, ^10^, ^11^ While our study was not specifically designed to examine OCT thinning in diabetes as a primary outcome measure, it is plausible that the functional pupillometric abnormalities observed in this study are due to retinal neural *dysfunction*, rather than frank structurally‐detectable *degeneration*, and may therefore not be reflected as RNFL/GCC thinning. Whether this dysfunction is therefore transient, or reversible with any therapeutic interventions, is an interesting area for future study. On the other hand, histological studies have demonstrated significant reduction in retinal ganglion cell and ipRGC density within the ganglion cell and inner plexiform layers of the retina in patients with DR, although no information on retinal layer thickness was presented.^45^ An alternative hypothesis is that early retinal ganglion cell or ipRGC loss may result in reductions in cellular density first, before affecting overall retinal layer thickness. This may mean that retinal dysfunction detected by chromatic pupillometry could be a more sensitive marker of early ipRGC loss than frank structural thinning of OCT layers, which may only occur in more severe or later stage disease. However, this hypothesis would have to be more rigorously evaluated with more detailed structural imaging studies.

In this study, we also report greater pupillometric dysfunction in a subset of diabetic patients with poorer glycemic control (HbA1c ≥ 8.0%) compared to those with better glycemic control (HbA1c < 8.0%). This appeared to be independent of DR status, as the proportion of patients with DR in each group was not significantly different. Reutrakul et al performed a small exploratory analysis which also showed a significant association between poorer glycemic control and lower PIPR amplitudes.^26^ However, this result did not take DR status into account. In contrast, Park et al did not find any significant correlation between HbA1c and pupillometric responses in their study.^25^ If there is truly a relationship between glycemic control and pupillometric dysfunction, this lends further support to the hypothesis that at least some of the retinal neural dysfunction we observe is potentially transient, or even reversible with treatment. A quick, convenient, non‐invasive assessment of glycemic control with a handheld device could have significant translational implications, but this relationship deserves further examination and validation in dedicated studies.

Though our study was not specifically aimed to evaluate the utility of HCP in detecting diabetes or DR in clinical settings, we show in exploratory analyses that HCP is able to classify patients with and without diabetes (AUC = 0.89), and with and without DR (AUCs between 0.72 and 0.88, depending on the population evaluated) with reasonable levels of diagnostic accuracy. The AUC value for classification of diabetic patients with and without DR was slightly lower at 0.72, but still reflects good performance despite the smaller sample sizes available for this particular analysis. The true clinical value of the device would depend largely on the specific clinical task and setting that it is applied to. Formal assessment of clinical utility and translation are beyond the scope of this study, and should ideally be carried out in a larger clinic‐based or community‐based study designed to address this question directly.

Our work has some limitations. First, the cross‐sectional nature of this study prevents us from drawing conclusions on the true temporal relationship between retinal neural dysfunction detected on pupillometry, and retinal vascular changes or DR. Second, the prognostic implications of the retinal neural dysfunction detected by chromatic pupillometry (and indeed DRN detected by other tests such as PERG, mfERG, perimetry or structural OCT) are still unclear. Some studies suggest that localised mfERG abnormalities can predict the location of eventual development of DR.^13^ Longitudinal studies are needed to validate the prognostic value of chromatic pupillometry (and other measures of DRN) towards meaningful clinical outcomes in diabetes. Third, we did not have information available on the duration of diabetes in our subjects, which could have been a confounding factor. Fourth, in defining poor glycemic control, we only had access to the most recent HbA1c values for some subjects. It would have been ideal if we had access to more HbA1c values over a longer period of time, to better define controlled and uncontrolled diabetes in our analyses. Fifth, although we did not detect any ophthalmoscopically visible DR in our DM without DR group, we are unable to exclude the presence of other early vascular changes that may be detectable on dye‐based angiography or OCT angiography.

In conclusion, significant retinal neural dysfunction can be demonstrated in diabetic patients using handheld chromatic pupillometry, even in the absence of clinically visible DR. In addition, pupillometric responses show greater reduction in patients with DR, and with poor glycemic control. Longitudinal studies could establish the possible prognostic value of such early retinal dysfunction in diabetes, and potentially validate pupillometric abnormalities as important biomarkers for ocular and systemic diabetes status.

## CONFLICT OF INTEREST

DM, RPN have a patent application based on the handheld pupillometer used in this study (PCT/SG2018/050204): Handheld ophthalmic and neurological screening device. No conflicting relationship exists for the other authors.

## Supporting information


**APPENDIX** **S1**: Supporting information.
