# Supplementary material for: Retinal neural dysfunction in diabetes revealed with handheld chromatic pupillometry
Source: Clin Exp Ophthalmol. 2022 Jun 13;50(7):745–56. doi: 10.1111/ceo.14116 (PMC9796882; doi:10.1111/ceo.14116)

SUPPORTING INFORMATION

Supplementary Table 1. Pupillometric parameters for controls and patients with diabetes without diabetic retinopathy (DM without DR), and controlled diabetes (glycated hemoglobin A1c < 8.0%). This additional analysis was performed for pupillometric parameters which were significantly different between controls and DM without DR, to ensure that the findings reported in the main manuscript were not confounded by glycemic control.

Pupillometric parameters	Controls (n = 93)	DM no DR, and HbA1c < 8.0% (n = 8)	P-value
Max blue, %, median (IQR)†	59.7 (6.1)	47.9 (8.2)	0.001
Max red, %, mean (SD)‡	55.6 (6.1)	43.8 (9.2)	<0.001
Blue PIPR 6s, %, mean (SD)‡	21.1 (6.4)	12.0 (5.7)	<0.001
Net PIPR 6s, %, mean (SD)§	4.5 (5.8)	1.6 (2.4)	0.01

Abbreviations:
DM = diabetes mellitus; DR = diabetic retinopathy; HbA1c = glycated hemoglobin A1c; max blue = baseline-adjusted maximal constriction amplitude to blue (469 nm) light; max red = baseline-adjusted maximal constriction amplitude to red (640 nm) light; blue PIPR 6s = post-illumination pupillary response assessed as the median constriction amplitude 6 seconds after blue light offset; net PIPR 6s = difference between blue PIPR 6s and red PIPR 6s

† Assessed using Mann-Whitney U test
‡ Assessed using Student's t-test
§ Assessed using Welch's t-test

Supplementary Table 2. Pupillometric parameters for controls and patients with diabetes, with and without diabetic retinopathy, with parameters associated with red light stimulus corrected for baseline pupil size prior to red light stimulus onset.

Pupillometric parameters	Controls (n = 93)	DM		P-value
		No DR (n = 25)	DR (n = 57)	
Baseline pupil size, mm, mean (SD)†	3.2 (0.6)	3.0 (0.7)	2.6 (0.6)	<0.001** ***
Phasic blue, %, mean (SD)‡	32.6 (6.9)	29.6 (9.4)	24.0 (8.5)	<0.001** ***
Phasic red, %, mean (SD)†	26.8 (6.9)	24.8 (6.9)	23.0 (8.3)	0.009**
Max blue, %, mean (SD)†	58.9 (5.2)	51.3 (8.6)	45.2 (9.9)	<0.001* ** ***
Max red, %, mean (SD)‡	48.6 (7.7)	42.9 (7.8)	37.8 (10.6)	<0.001* ** ***
Blue PIPR 6s, %, mean (SD)‡	21.1 (6.4)	16.4 (6.8)	14.2 (6.5)	<0.001* **
Red PIPR 6s, %, median (IQR)§	10.0 (8.4)	9.8 (5.2)	8.1 (8.9)	0.13
Net PIPR 6s, %, median (IQR)§	10.1 (9.1)	6.1 (7.0)	5.7 (7.7)	<0.001* **

Abbreviations:
DM = diabetes mellitus; DR = diabetic retinopathy; phasic red = median baseline-adjusted pupil constriction 0.5-2.5s following red (640 nm) light onset; max red = baseline-adjusted maximal constriction amplitude to red light; red PIPR 6s = post-illumination pupillary response assessed as the median constriction amplitude 6 seconds after red light offset; net PIPR 6s = difference between blue PIPR 6s and red PIPR 6s

† Assessed using linear regression with age as a covariate
‡ Assessed using One-way ANOVA followed by post hoc Tukey tests
§ Assessed using Kruskal-Wallis followed by post hoc Dunn's tests
* Significant difference between Controls vs No DR
** Significant difference between Controls vs DR
*** Significant difference between No DR vs DR

Supplementary Table 3. Pupillometric parameters for patients with diabetes, stratified based on glycemic control, with parameters associated with red light stimulus corrected for baseline pupil size prior to red light stimulus onset.

Pupillometric parameters	HbA1c < 8.0% (n = 38)	HbA1c ≥ 8.0% (n = 19)	P-value
Baseline pupil size, mm, mean (SD)†	2.9 (0.6)	2.4 (0.6)	0.01
Phasic blue, %, mean (SD)†	23.9 (7.5)	25.0 (9.9)	0.65
Phasic red, %, mean (SD)†	23.4 (7.0)	22.8 (10.0)	0.81
Max blue, %, mean (SD)†	48.2 (8.0)	41.8 (11.5)	0.02
Max red, %, mean (SD)†	40.4 (8.6)	34.5 (11.9)	0.04
Blue PIPR 6s, %, mean (SD)†	14.6 (6.0)	12.5 (8.2)	0.28
Red PIPR 6s, %, mean (SD)‡	8.6 (5.4)	7.0 (9.1)	0.49
Net PIPR 6s, %, mean (SD)§	6.0 (5.1)	5.5 (8.0)	0.80

Abbreviations:
HbA1c = glycated hemoglobin A1c; phasic red = median baseline-adjusted pupil constriction 0.5-2.5s following red (640 nm) light onset; max red = baseline-adjusted maximal constriction amplitude to red light; red PIPR 6s = post-illumination pupillary response assessed as the median constriction amplitude 6 seconds after red light offset; net PIPR 6s = difference between blue PIPR 6s and red PIPR 6s

† Assessed using Student's t-test
‡ Assessed using Welch's t-test
§ Assessed using linear regression with age as a covariate

Supplementary Figure 1. Relative contributions of pupillometric parameters to gradient boosting machines used to distinguish (A) controls (n = 93) versus diabetic patients without or with DR (DM without DR, and DR; n = 82), (B) patients without DR (controls, and DM without DR; n = 118) versus patients with DR (n = 57), and (C) diabetic patients without DR (DM without DR; n = 25) versus diabetic patients with DR (n = 57). The most important pupillometric parameters for each classification problem were used to build a logistic regression model. Abbreviations: GLM = generalized linear model; phasic blue = median baseline-adjusted pupil constriction 0.5-2.5s following blue (469 nm) light onset; phasic red = median baseline-adjusted pupil constriction 0.5-2.5s following red (640 nm) light onset; max blue = baseline-adjusted maximal constriction amplitude to blue light; max red = baseline-adjusted maximal constriction amplitude to red light; blue PIPR 6s = post-illumination pupillary response assessed as the median constriction amplitude 6 seconds after blue light offset; red PIPR 6s = post-illumination pupillary response assessed as the median constriction amplitude 6 seconds after red light offset; net PIPR 6s = difference between blue PIPR 6s and red PIPR 6s.

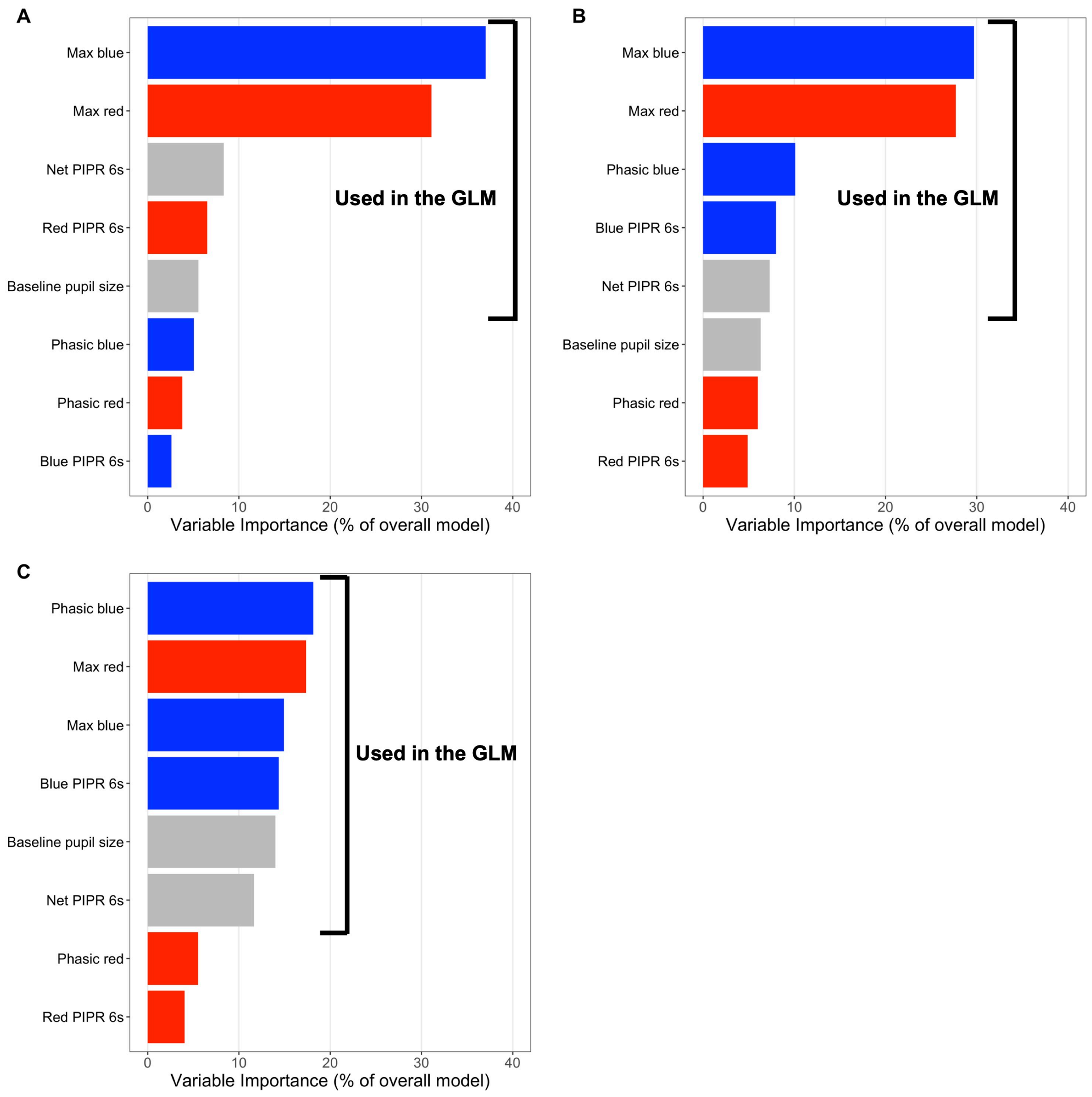

Supplement: Supplementary file 1 — APPENDIX S1: Supporting information. [file CEO-50-745-s001.pdf]
